# The novel genus *Brakhagea* gen. nov. is constituted by four planctomycetal species isolated from aquatic environments in Northern Germany

**DOI:** 10.1038/s41598-026-47393-x

**Published:** 2026-04-19

**Authors:** Gaurav Kumar, Nicolai Kallscheuer, Jonathan Hammer, Tom Haufschild, Christian Jogler

**Affiliations:** 1 Department of Microbial Interactions, Institute of Microbiology, Friedrich Schiller University, Jena, Germany; 2 Cluster of Excellence Balance of The Microverse, Friedrich Schiller University, Jena, Germany

**Keywords:** *Planctomycetota*, Schlesner strain collection, *Aureliella helgolandensis*, *Pirellulaceae*, Ecology, Ecology, Genetics, Microbiology, Molecular biology

## Abstract

Four budding and pink-pigmented planctomycetal strains, designated SH449^T^, SH551^T^, SH467^T^, and SH501^T^, were isolated from diverse environmental sources, including Fjord Schlei (a 42 km brackish arm of the Baltic Sea), ponds and wastewater lagoons in Northern Germany, as part of an isolation campaign led by Heinz Schlesner at Kiel University between the 1980s and early 2000s. Analyses based on five phylogenetic markers indicate that these strains constitute a novel genus within the family *Pirellulaceae* of the phylum *Planctomycetota*. All four strains are aerobic, heterotrophic, mesophilic, and exhibit neutrophilic properties. Strains SH551^T^ and SH501^T^ possess at least one plasmid that is not present in the other two strains. Genome analyses revealed that their genomes are of comparable size to those of other members of the *Pirellulaceae* family, exhibiting a DNA G + C content ranging from 49 to 54%. Analysis of predicted biosynthetic gene clusters shows that all four strains possess the genetic potential to produce bioactive compounds and may therefore be regarded as putative “talented producers”. The polyphasic approach places all four novel strains in a new genus within the family *Pirellulaceae*, for which the name *Brakhagea* gen. nov. is proposed. The novel isolates represent four distinct novel species, *Brakhagea baltica* sp. nov. (type strain SH449^T^ = KCTC 102025^T^ = DSM 116586^T^), *Brakhagea lacunae* sp. nov. (type strain SH551^T^ = KCTC 102023^T^ = DSM 116785^T^ = CECT 30846^T^), *Brakhagea slesvicensis* sp. nov. (type strain SH467^T^ = KCTC 102026^T^ = DSM 116378^T^), and *Brakhagea aquatica* sp. nov. (type strain SH501^T^ = DSM 102088^T^ = CECT 30906^T^).

**Supplementary Information:**

The online version contains supplementary material available at 10.1038/s41598-026-47393-x.

## Introduction

*Planctomycetota*, a bacterial phylum within the *Planctomycetota–Verrucomicrobiota–Chlamydiota* (PVC) superphylum^1^, comprises Gram-negative bacteria with uncommon morphological and physiological characteristics that differentiate them from other bacterial taxa. These characteristics include diverse reproductive morphotypes, such as budding or binary fission, and the absence of the otherwise ubiquitous bacterial cell division protein FtsZ^2–5^. Some planctomycetes known as ‘bacteria of prey’ are thus far the only bacteria employing phagocytosis-like cell engulfment to eat other bacteria alive^6,7^. Members of the phylum *Planctomycetota* are characterized by a highly condensed nucleoid, the presence of cytoskeleton-like elements, extensive cytoplasmic membrane invaginations, and complex dimorphic life cycles^4^. The life cycle observed in many strains starts from a sessile mother cell that is attached to a surface via a holdfast structure^8^. Subsequently, a flagellated daughter cell originates from the mother cell, grows and eventually pinches off from the mother cell^9^. Morphologically, planctomycetal colonies display a wide spectrum of colors, ranging from white and beige to pink, red, or orange^10^, while individual cells display a variety of morphologies ranging from spherical to rod, ovoid, or pear-shaped forms^11^. Cells often feature crateriform structures on their cell surface with pili-like fibre structures around or associated with them. However, their fastidious nature poses significant challenges for isolation using standard microbiological media, as they are frequently outcompeted by faster-growing bacteria. The distinctive cellular biology of planctomycetes is complemented by yet untapped metabolic functions, which offer significant potential for biotechnological applications. Recent studies have demonstrated that members of the phylum display antimicrobial activity, probably by the biosynthesis of small molecules^4,12^. Relative to other bacterial phyla, planctomycetes can have large genomes of up to 12.4 Mbp^13^. Furthermore, this phylum is distinguished by containing the highest proportion of predicted genes with unknown functions^4^. This is partly due to the only recent development of genetic tools for the phylum *Planctomycetota*^14,15^.

Planctomycetes exhibit remarkable metabolic versatility regarding the utilization of carbon and energy sources including complex polysaccharides, enabling them to colonize a diverse array of ecological niches^4^. Most of the characterized strains so far are aerobic, mesophilic, and heterotrophic, thriving in environments with acidic to alkaline pH. However, certain strains, notably the members of the class “*Candidatus* Brocadiia”, follow a specialized metabolism involving the anaerobic oxidation of ammonium (anammox)^16^. Representatives of the described classes of the phylum have been identified across a broad spectrum of terrestrial habitats, including soils^17,18^ and peat bogs^19^, as well as aquatic environments such as marine waters^20–23^, freshwater systems^24,25^, sediments^26–28^, and deep-sea deposits^29^. Some strains are even capable of inhabiting extreme environments, including hot springs^30^, and desert soils^31^ or marine volcanic areas^32–38^, underscoring their exceptional adaptability to different ecological conditions. Additionally, in numerous instances members have been documented in (symbiotic) association with other organisms, including sponges^22,39–43^, jellyfish^44^, phototrophs^11,45–51^, and the gastrointestinal tract^52^. However, besides biotic surfaces, many members of the phylum *Planctomycetota* attach to abiotic surfaces as well if incubated in marine or limnic habitats^46,50,53,54^.

In this study, we describe a novel genus that includes four new species within the family *Pirellulaceae*. According to the List of Prokaryotic Names with Standing in Nomenclature (LPSN), the current family *Pirellulaceae* (as of March 2026) encompasses 18 genera validly published under the International Code of Nomenclature of Prokaryotes (ICNP) and includes the highest number of described species within the entire phylum. The isolates presented here belong to the strain collection of Heinz Schlesner (“SH” strains, named after his initials), a pioneering researcher of budding bacteria, including *Planctomycetota*, at Kiel University, Germany. Over the last two decades of the 20th century, he isolated hundreds of novel strains from various locations in Northern Germany and world-wide. Following Schlesner’s retirement in July 2002, the collection was transferred to the research group led by Michael Thomm at the Institute for Biochemistry, Genetics, and Microbiology at the University of Regensburg. In 2019, the collection of 500 strains was relocated to the Department of Microbial Interactions at Friedrich Schiller University Jena^55^. Advances in genome sequencing compared to the end of the last century will now enable the characterization of the novel taxa in this collection based on high-quality genome sequences in addition to the analysis of phenotypic features. The phylogenetic inference is mainly based on the analysis or single gene- and whole-genome-based phylogenetic markers that turned out to have a better resolution compared to classical chemotaxonomic analyses when applied for strains belonging to the phylum *Planctomycetota*^39^.

## Materials and methods

### Strains, cultivation and 16S rRNA gene sequences

All four strains, SH449^T^, SH501^T^, SH551^T^, and SH467^T^ were isolated from distinct water bodies in Northern Germany by Heinz Schlesner. Strain SH449^T^ was obtained from Fjord Schlei, a 42 km brackish arm of the Baltic Sea, strain SH501^T^ from surface water at a lagoon in Schrevenpark pond in Kiel, strain SH551^T^ from a village pond in Kopendorf, Fehmarn Island and strain SH467^T^ from the wastewater aeration lagoon at a former sugar processing plant in Schleswig. Detailed methodologies and findings related to their isolation are documented in studies conducted under the supervision of Heinz Schlesner^56,57^. Cultivation of the strains was performed using two distinct media: M30PY for strains SH449^T^ and SH467^T^, and M1aPY for strains SH501^T^ and SH551^T^. The preparation of these media followed previously established protocols^58^. The designation “PY” denotes the supplementation of 0.25 g/L peptone and 0.25 g/L yeast extract in the media. Solid media were prepared with 15 g/L agar that was autoclaved separately in a volume of 200 mL distilled water and added to the autoclaved medium prior to pouring of the plates. The 16S rRNA genes of all four isolates were amplified via polymerase chain reaction (PCR), purified using a standardized workflow^59^, and subsequently sequenced at Macrogen Europe (Amsterdam, The Netherlands).

### Physiological analyses

To ascertain the optimal temperature for microbial growth, 100 µL of supernatant from an exponentially growing culture, devoid of visible aggregates, was spread on agar plates. These plates were incubated in duplicates at temperatures ranging from 4 °C to 42 °C. Daily inspections were conducted, and growth was assessed by determining the time required for the formation of visible colonies or lawns. The temperature at which colonies or lawns first appeared was identified as the optimal growth temperature. The optimal pH for growth was determined in a two week 96-well plate reader experiment with continuous shaking. Media were supplemented with 100 mM of one of the following buffering agents: 2-(*N*-morpholino)ethanesulfonic acid (MES) for pH 5.0 and 6.0, 4-(2-hydroxyethyl)−1-piperazineethanesulfonic acid (HEPES) for pH 7.0, 7.5, and 8.0, or *N*-cyclohexyl-2-aminoethanesulfonic acid (CHES) for pH 9.0 and 10.0. Growth was quantified by measuring the optical density at 600 nm (OD_600_) using a BioTek Epoch2 microplate spectrophotometer (Agilent) at 24–28 °C depending on the determined temperature optimum of the strain. Each condition was tested in duplicates. Each measurement cycle, lasting 30 min, comprised two shaking phases of 15 min interrupted by the OD_600_ measurements of the entire 96-well plate (Brand Plate pureGrade™ S, transparent sterile 96-well plates). The shaking regime of the 15 min interval was changed between linear to orbital to double-orbital every 5 min. To mitigate condensation on the plate lid, a temperature gradient was established, maintaining the liquid at 24–28 °C (depending on the temperature optimum of the strain) and the lid temperature was maintained at a 2 °C higher temperature. For data analysis, the mean values of each time point were calculated, and the mean of the medium blank was subtracted. The growth rate at each pH was calculated from a selection of data points with the maximal slope of the natural logarithm of the OD_600_ values plotted against the cultivation time.

### Light microscopy and cell size determination

Light microscopy was performed following the methodology outlined in a previous study^60^. In brief, cells harvested from liquid cultures at the half-maximal OD_600_ were mounted on a 1% (w/v) agarose cushion prepared in deionized water (dH_2_O). Once the culture had dried on the agarose cushion, a coverslip was applied and secured at the edges with VLAP (a 1:1:1 mixture of vaseline, lanolin, and paraffin by weight) to ensure stability. Imaging was conducted using an inverted Nikon Ti2 microscope equipped with a Nikon Plan Apo λ 100x immersion oil objective, configured with a phase ring for phase-contrast (PhC) imaging or without for differential interference contrast (DIC) imaging^61^. The system included a Nikon DS-Ri2 camera and NIS-Elements software (version 5.30). Three-channel RGB images were processed in FIJI^62^ to generate single-channel RGB images. TIFF files were subsequently analyzed in BacStalk^63^, with cell segmentation performed using thresholds of 25 pixels for cell size and 15 pixels for minimum cell size. A total of three biological replicates, each comprising 150 cells, were evaluated. For data visualization, results were uploaded to SuperPlotsOfData^64^. To enhance visualization, brightness and contrast adjustments were manually applied to PhC and DIC images.

### Genomic DNA isolation, genome sequencing, annotation and analysis

Genomic DNA extraction, and quality control were conducted following established protocols^60^. *De novo* genome assembly was performed using long-read data from Oxford Nanopore, with subsequent polishing utilizing short-read data from Illumina sequencing. Details on the sequencing chemistry and bioinformatic workflow as well as tools, tool versions and optional parameters are stated in Table S1. Illumina sequencing was performed by Eurofins Genomics (Ebersberg, Germany). The genome completeness was assessed with BUSCO (version 5.8.2), while coding density and DNA G + C content were evaluated using CheckM (version 1.2.3). Following an initial annotation with Prokka (version 1.14.5), the chromosome was re-oriented to the start codon of the *dnaA* gene encoding the replication initiator protein, and subjected to final re-annotation using PGAP (version 2025-05−06, build 7983).

### Nucleotide sequence accession numbers

The 16S rRNA gene sequences were deposited in the GenBank database under the following accession numbers: PV955651 (SH449^T^), PV955658 (SH551^T^), PV955674 (SH467^T^) and PV955675 (SH501^T^). Genome sequence information is available from NCBI under the accession numbers: SH449^T^: CP197409; SH551^T^: CP197424 (chromosome) and CP197425 (plasmid pSH551_1); SH467^T^: CP197412; SH501^T^: CP197422 (chromosome) and CP197423 (plasmid pSH501_1).

### Phylogenetic and genome-based analyses

The full-length 16S rRNA gene sequences of the novel planctomycetal isolates were retrieved from the genomes annotated with Prokka and employed to identify the closest relatives via NCBI BLAST. Maximum-likelihood phylogenetic trees were constructed based on 16S rRNA gene sequences and multi-locus sequence analysis (MLSA) for the novel strains and type strains of all species within the phylum *Planctomycetota*, with the MLSA-based tree limited to strains belonging to the family *Pirellulaceae*. The 16S rRNA gene sequences of the type strains of *Opitutus terrae* (NCBI accession no. AJ229235), *Kiritimatiella glycovorans* (accession no. NR_146840), and *Lentisphaera araneosa* (accession no. NR_027571), representing members of the *Planctomycetota-Verrucomicrobiota-Chlamydiota* (PVC) superphylum outside of the phylum *Planctomycetota*, were used as outgroup. Sequence alignments were conducted using ClustalW^65^, and phylogenetic trees were reconstructed with FastTree v2.2^66^ employing 1000 bootstrap replicates. The MLSA-based phylogeny was inferred using the autoMLST tool^67^ with 500 bootstrap replicates, including the genomes of *Planctopirus limnophila* DSM 3776^T^ (GenBank acc. no. CP001744.1), *Gimesia maris* DSM 8797^T^ (GenBank acc. no. CP042910.1) and *Planctomicrobium piriforme* DSM 26348^T^ (GenBank acc. no. GCA_900113665.1), all from the family *Planctomycetaceae*, as outgroup. Phylogenetic trees were visualized using iTOL v6^68^. A 16S rRNA gene sequence similarity matrix was generated using TaxonDC^69^ based on the ClustalW alignment used for phylogenetic tree construction. Average amino acid identities (AAI) and average nucleotide identities (ANI) were calculated using scripts from the enveomics collection^70^. Additional phylogenetic markers, including *rpoB* gene sequence similarity and percentage of conserved proteins (POCP), were determined following established methods^71,72^. The pangenome of selected strains was constructed using anvi’o v.8 with default parameters^73^. Biosynthetic gene clusters (BGCs) were predicted with antiSMASH v.8.0^74^ in relaxed detection mode and with all extra features activated, and carbohydrate-active enzymes (CAZymes) were identified using dbCAN3^75^.

## Results and discussion

### Phylogenetic inference

**Fig. 1:**
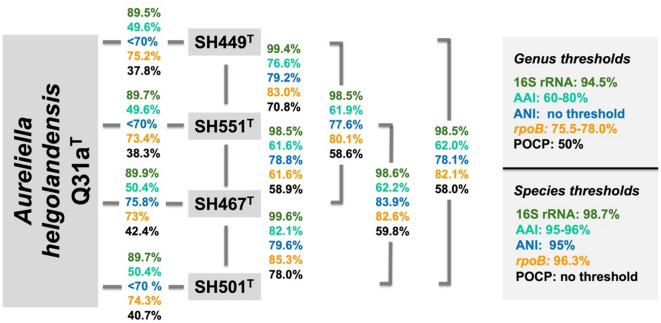
Comparison of phylogenetic markers for genus and species delineation. Markers used: 16S rRNA gene sequence identity (16S rRNA), average amino acid identity (AAI), average nucleotide identity (ANI), sequence similarity of a partial sequence of the *rpoB* gene *(rpoB)*, percentage of conserved proteins (POCP).

Initial nucleotide BLAST analyses of the 16S rRNA gene sequences of the four strains SH449^T^, SH551^T^, SH467^T^, and SH501^T^ demonstrated the highest sequence similarity (< 90%) to *Aureliella helgolandensis* Q31a^T^ in the family *Pirellulaceae* (Fig. 1). This level of similarity is substantially below the established 94.5% threshold for 16S rRNA gene sequence similarity used to delineate a new genus^72^, indicating a genus-level relationship within the *Pirellulaceae* family. Furthermore, comparisons of POCP and AAI values between the four isolates and *A. helgolandensis* Q31a^T^ revealed maximum similarities of 42.4% and 50.4%, respectively. These values fall well below the recognized thresholds of 50% for POCP and 60% for AAI used for genus delineation^72^, thereby providing robust support for the proposal of a novel genus (Fig. 1).

**Fig. 2:**
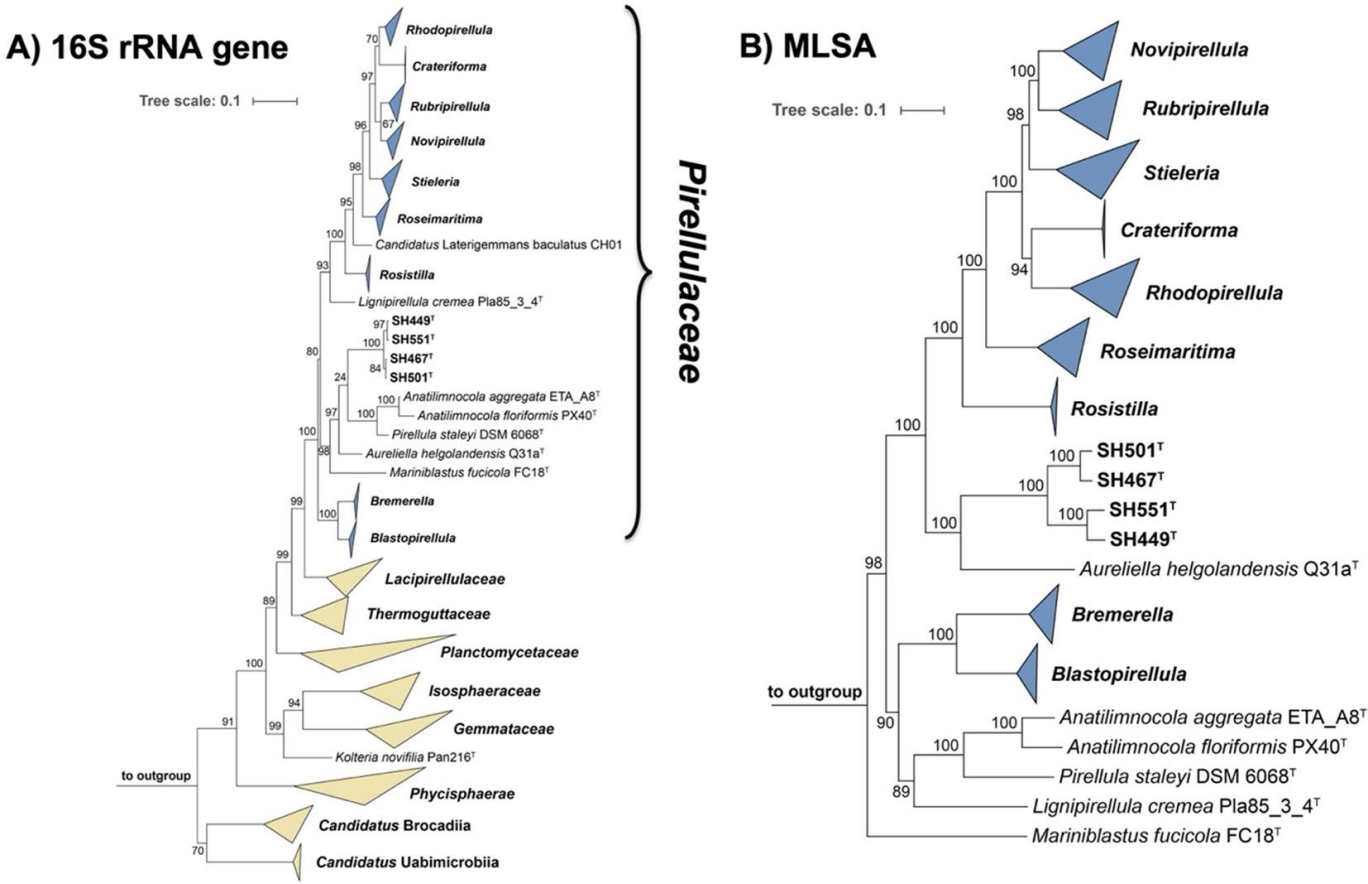
Phylogenetic placement. **(A)** Maximum likelihood phylogenetic tree based on 16S rRNA gene sequences showing the phylogenetic relationship of the novel isolates and other members of family *Pirellulaceae*. Bar, 0.1 substitutions per nucleotide position. **(B)** Multi-locus sequence analysis (MLSA)-based phylogenetic tree constructed with the genomes of characterized members in the family *Pirellulaceae*. The tree was computed based on a set of at least 30 single-copy gene-encoding proteins in a maximum likelihood approach with 500 bootstrap replications. Bar, 0.1 substitution per amino acid position. Bootstrap values for both trees are given at the nodes (in %). Phylogenetic trees were visualized with iTOL v6. In both trees, the collapsed clade *Rhodopirellula* includes the genera *Allorhodopirellula*, *Aporhodopirellula* and *Neorhodopirellula* (that we do not regard as distinct genera), while the collapsed clade *Stieleria* includes the genus *Roseiconus* (the *Roseiconus* species were recently re-named *Stieleria*). NCBI RefSeq accession numbers of the genome sequences used for tree reconstruction are provided in Table S2.

The 16S rRNA gene sequence similarity between strains SH449^T^ and SH551^T^ is 99.4%, while that between strain SH467^T^ and SH501^T^ is 99.6%, both exceeding the 98.7% threshold typically used to delineate bacterial species on the basis of 16S rRNA gene sequence similarity^72^(Fig. 1). Both phylogenetic trees show clustering patterns that reflect these similarities (Fig. 2). However, the MLSA-based phylogenetic tree suggests more distant clustering; with SH449^T^ and SH551^T^ forming a closer-related clade separate from the clade of SH467^T^ and SH501^T^ (Fig. 2). In a comparative analysis of phylogenetic markers between strains SH449^T^ and SH551^T^, ANI, AAI, and *rpoB* gene sequence similarity values were determined to be 79.2%, 76.6%, and 83.0%, respectively. Likewise, for strains SH467^T^ and SH501^T^, the corresponding ANI, AAI, and *rpoB* similarity values were 79.6%, 82.1%, and 85.3%, respectively. These values fall substantially below the established thresholds for species delineation, which are 95% for ANI, 95–96% for AAI, and 96.3% for *rpoB*^71,72^(Fig. 1). Consequently, these findings provide robust evidence that strains SH449^T^, SH551^T^, SH467^T^, and SH501^T^ represent four distinct novel species. Comparative analysis of the clades encompassing SH449^T^/SH551^T^ and SH467^T^/SH501^T^ indicates notably high POCP (> 50.0%) and AAI (> 60.0%) values, clearly confirming that all four novel isolates belong to the same novel genus. Similar observations that strains belong to separate species despite 16S rRNA gene sequence similarities exceeding the threshold occur occasionally in the phylum *Planctomycetota.* The 16S rRNA gene sequence identity of *Planctopirus limnophila* Mü290^T^ and *Planctopirus ephydatiae* spb1^T^ is 99.99% and chemotaxonomic measures fell short in distinguishing the two species. Only the analysis using whole genome-based phylogenetic markers revealed that they indeed represent two separate species^39^.

### Genomic characteristics

In comparison to the genome of *A. helgolandensis* Q31a^T^, which has a size of 8.44 Mbp, the genomes of the four novel isolates are notably smaller, with strain SH467^T^ exhibiting a genome size of only 5.87 Mbp (Table 1). Strains SH551^T^ and SH501^T^ harbor a plasmid, whereas strains SH449^T^ and SH467^T^ lack any extrachromosomal elements. The DNA G + C content of the novel strains ranges from 49.4 to 54.2%, which is slightly below the value obtained for the genome of *A. helgolandensis* Q31a^T^ (55.3%). In terms of numbers of protein-coding genes per Mbp, relative numbers of hypothetical protein-encoding genes, coding density, and numbers of tRNA genes, the genomes of strains SH449^T^ and SH551^T^ exhibit only minor differences when compared to the more pronounced differences observed between strains SH467^T^ and SH501^T^ (Table 1). Across all four strains, no variation in the copy numbers of 5S, 16S, and 23S rRNA genes can be observed, which are consistently present in single copy per genome. In contrast, in *A. helgolandensis* Q31a^T^, these genes are present in two copies each. Among the novel strains, strain SH501^T^ exhibits the highest tRNA gene count, with 81 tRNA genes, while strain SH467^T^ harbours only 61 tRNA genes.

**Table 1:** Comparison of genomic and genome-encoded features of strains SH449^T^, SH551^T^, SH467^T^, SH501^T^, and *Aureliella helgolandensis* Q31a^T^.

Characteristics	*Aureliella helgolandensis* Q31a^T^	SH449^T^	SH551^T^	SH467^T^	SH501^T^
**Genomic features**					
Genome size (bp)	8,439,957	7,050,144	7,251,192	5,868,335	6,701,825
Contigs	1 (closed)	1 (closed)	2 (closed)	1 (closed)	2 (closed)
Plasmids	0	0	1	0	1
DNA G + C (%)	55.3	51.6	49.4	54.2	53.1
Genes	6049	5480	5481	4426	5365
Genes/Mbp	717	777	756	754	801
Protein-coding genes	5921	5387	5383	4337	5239
Protein-coding genes/Mbp	702	764	742	739	782
Hypothetical proteins*	1484	1517	1500	1005	1526
Hypothetical proteins (%)	25.1	28.2	27.9	23.2	29.1
Coding density (%)	84.7	88.7	88.1	89.7	88.9
rRNA genes (5S,16S,23S)	2, 2, 2	1, 1, 1	1, 1, 1	1, 1, 1	1, 1, 1
tRNA genes	72	62	66	61	81
**Secondary metabolite-associated biosynthetic gene clusters**					
Terpene	3	3	3	2	2
Terpene Precursor	2	2	2	2	2
Type I polyketide synthase	0	2	2	1	1
Type III polyketide synthase	1	0	1	1	1
NRPS-Like	2	2	2	1	2
RiPP-Like	1	0	0	0	0
Resorcinol	1	0	2	0	0
Indole	0	0	0	0	1
Acyl amino acid	1	0	0	0	0
Total number of BGCs	11	9	12	7	9
**Carbohydrate-active enzymes**					
Glycoside hydrolases	66	41	42	38	38
Glycosyltransferases	69	51	60	51	52
Polysaccharide lyases	8	1	1	4	2
Carbohydrate esterases	27	17	17	17	16
Carbohydrate-bind. modules	17	13	21	14	15
Auxiliary activities	4	2	2	3	3
CAZyme genes (total)	191	125	143	127	126
CAZyme genes per Mbp	23	18	20	22	19

*based on the PGAP-annotated genomes.

### Pangenome reconstruction and evaluation of genome-encoded functions

**Fig. 3:**
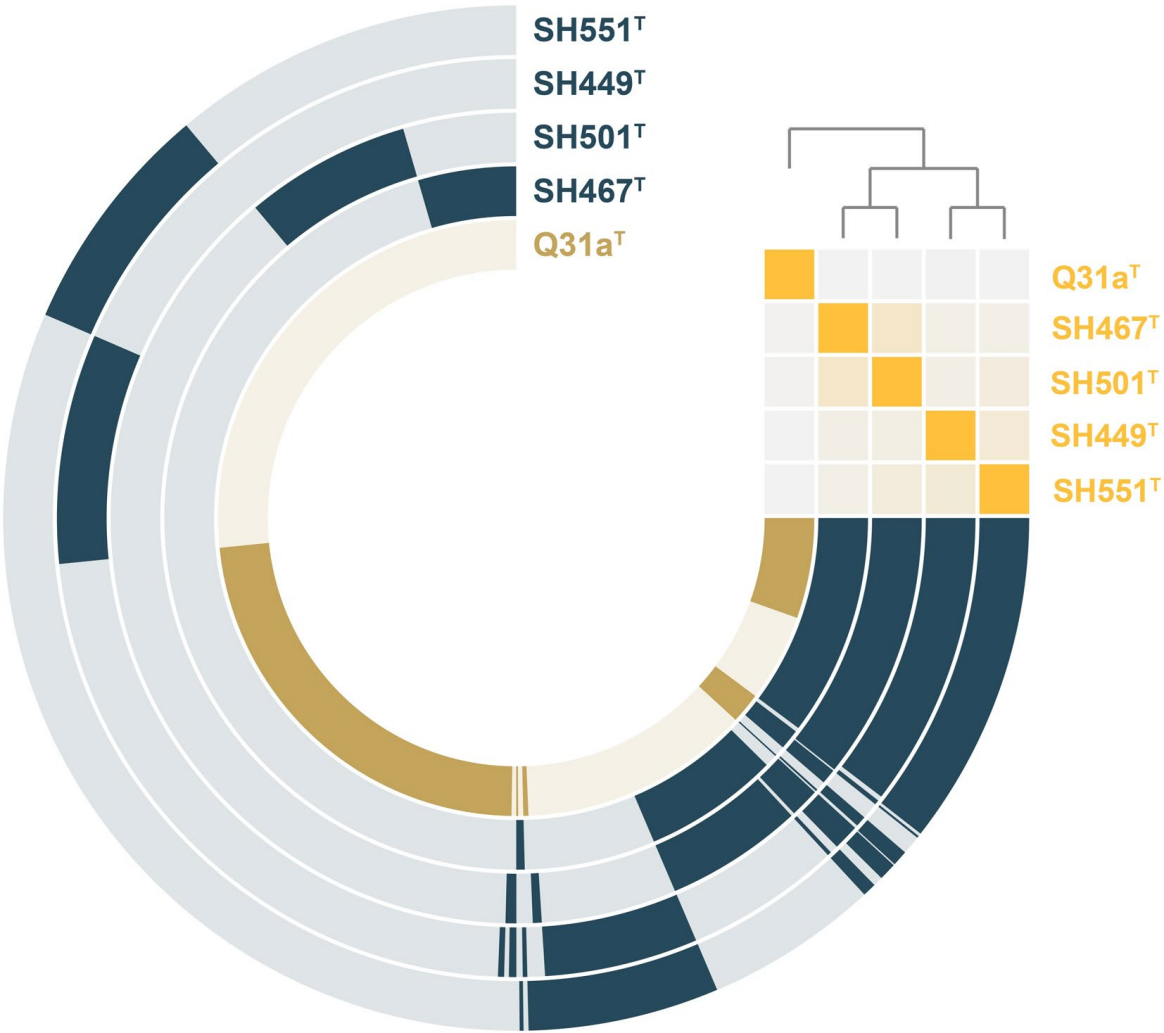
Pangenome reconstruction. Each open circle represents the pangenome of all strains but is colored darker when the gene is present in the respective genome. The heatmap in the upper right corner indicates the degree of relationship based on ANI values (ANI ≤ 70%, pale orange to ANI = 100%, bright orange).

To visualize the genome-based similarity among the compared strains, a pangenome was constructed and analyzed. This pangenome, encompassing the genomes of all five species including *A. helgolandensis* Q31a^T^, comprises 14,479 genes, with 1,057 genes being conserved across all five strains (core genome) and 2,038 genes being conserved among the four novel isolates (Fig. 3). The remaining genes are either unique to individual strains (singletons) or not conserved across all strains. The singleton gene count for strains SH449^T^, SH551^T^, SH467^T^, and SH501^T^ is 1588, 1440, 869, and 1308, respectively. Beyond the core genome (represented in the 3–4 o’clock segment in the pangenome visualization), the isolates SH467^T^ and SH501^T^ share additional genes (5 o’clock in the pangenome visualization). Likewise, the other two isolates SH449^T^, and SH551^T^, share separate additional genes (6 o’clock in the pangenome visualization). These findings align with the close species-level relationship among the novel isolates and their more distant relationship to *A. helgolandensis* Q31a^T^. Smaller genomes, like that of strain SH467^T^ (5.8 Mbp), which likely encode fewer accessory functions compared to many other members in the class *Planctomycetia* (genome sizes of up to 12.4 Mbp), are vital for pinpointing conserved, yet uncharacterized genes in future studies.

Genome mining for secondary metabolite-associated biosynthetic gene clusters (BGCs) using antiSMASH identified 7–12 BGCs each in the genomes of the four novel isolates (Table 1). Of these, four to five BGCs are likely associated with terpenoid or terpenoid precursor biosynthesis. Other predicted BGCs include genes encoding putative type I or type III polyketide synthases or are linked to the synthesis of short peptides formed by non-ribosomal peptide synthetase (NRPS)-like enzymes. The putative synthesis of resorcinols is unique to strain SH551^T^, while synthesis of indoles is specific to strain SH501^T^. Across all four novel isolates, the identification of CAZyme-encoding genes resulted in 120–200 hits per genome, with comparable numbers of hits in the classes of glycoside hydrolases and glycosyltransferases, though these numbers were lower than in *A. helgolandensis* Q31a^T^ (Table 1). This is not surprising since the genome of *A. helgolandensis* Q31a^T^ is at least 1 Mbp larger than that of the newly identified isolates. The counts of genes encoding polysaccharide lyases or proteins with auxiliary activities fall below ten per genome for all five genomes analyzed. This indicates that all five strains can in principle degrade complex carbohydrates into simpler sugars, however, the exact substrate spectrum should be determined in future cultivation experiments and cannot be predicted from the genome-based analysis. Strains SH449^T^ and SH467^T^ possess a single CRISPR array each with 34 and 45 spacers, respectively. Genes encoding phage-derived components (e.g., phage tail tape measure, minor head, and tail tube proteins) suggest prophage regions in the genomes. Conversely, the novel isolates SH551^T^ and SH501^T^ also contain genes encoding putative phage proteins but lack CRISPR arrays. The here described set of closely related strains could be valuable for investigating defense mechanisms or phage-host co-evolution in the phylum *Planctomycetota*. Noteworthy, the presence of plasmids in the two strains SH551^T^ and SH501^T^ that lack CRISPR array might suggest a potential functional relationship worth addressing further.

### Physiological characterization

**Fig. 4:**
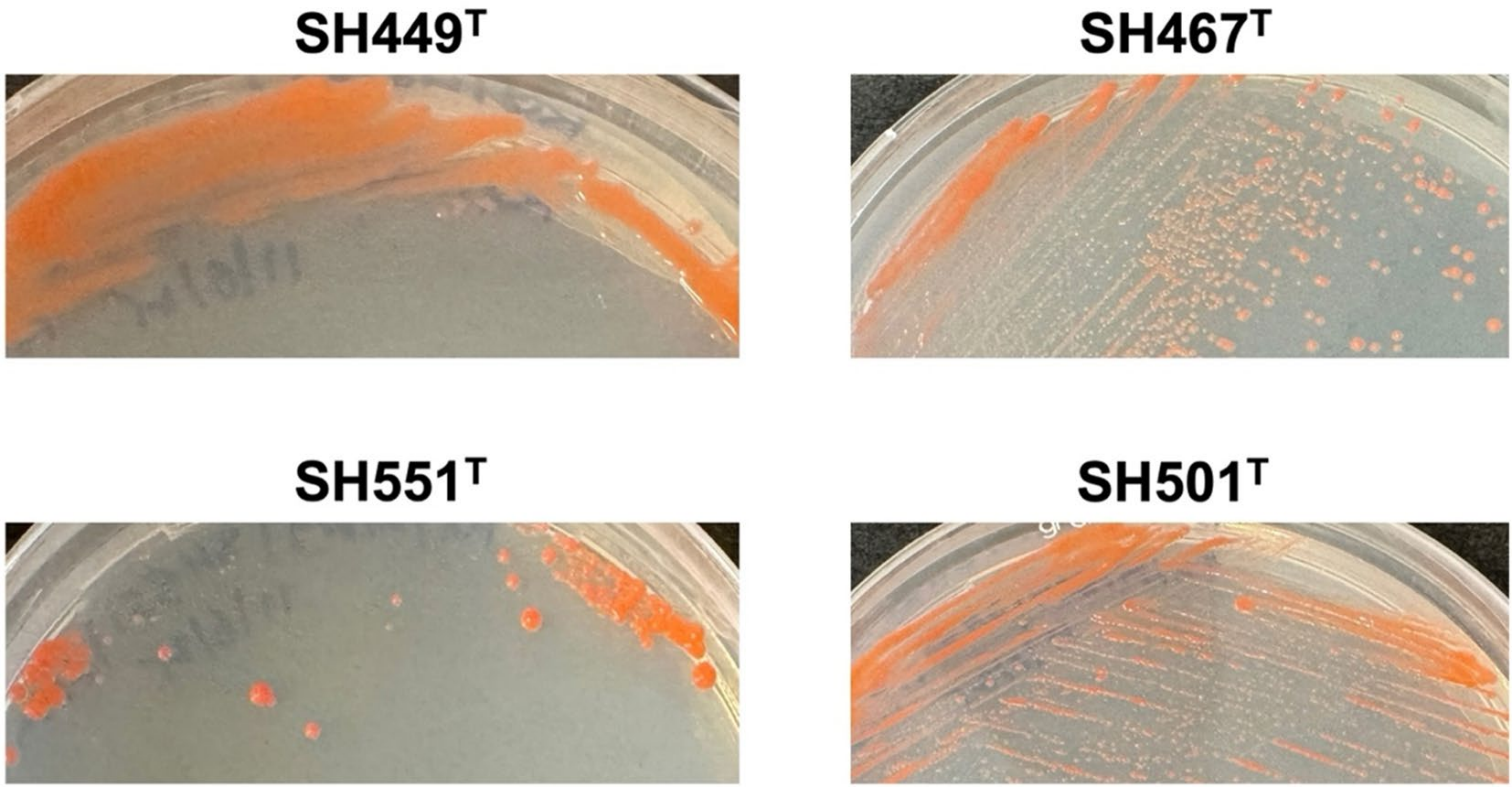
Appearance of colonies on plates. Colonies of the four strains have the same color, but colonies differ in size, shape and consistency. Strains SH467^T^ and SH501^T^ form smaller circular colonies, whereas colonies of strains SH449^T^ and SH551^T^ are much bigger, irregular and have a slimier consistency.

In both, agar plate and liquid cultures, all four strains exhibit light to dark pink pigmentation (Fig. 4), contrasting the lucid white appearance of their closest relative, *A. helgolandensis* Q31a^T^. Strains SH551^T^, SH467^T^, and SH501^T^ produce circular, convex colonies characterized by entire margins. In contrast, colonies of strain SH449^T^ exhibit either circular or irregular morphologies and a more mucoid consistency (Fig. 4). Strain SH449^T^ exhibits growth within a temperature range of 18–32 °C, with an optimum at 28 °C, and demonstrates tolerance to alkaline conditions up to a pH of 10.0, with an optimal pH of 8.0. In contrast, strain SH551^T^ displays growth between 21 and 28 °C, with an optimum at 28 °C, and prefers a pH of 7.5 for optimal growth (Table 2). Strains SH467^T^ and SH501^T^ demonstrate growth at temperatures up to a maximum of 37 °C, with an optimal growth temperature of 28 °C. Both, strains SH467^T^ and SH501^T^, exhibit optimal growth at a pH of 7.5. Additionally, strain SH467^T^ is capable of biomass formation at an alkaline pH of 10.0 (Table 2). The aerobic heterotrophic lifestyle of *A. helgolandensis* Q31a^T^ is also followed by the four novel strains.

**Table 2:** Comparison of phenotypic characteristics of strain SH449^T^, SH551^T^, SH467^T^, SH501^T^ to their closest relative *Aureliella helgolandensis* Q31a^T^.

Characteristics	*Aureliella helgolandensis* Q31a^T^	SH449^T^	SH551^T^	SH467^T^	SH501^T^
**Sampling Information**					
Location	Helgoland, Germany	Baltic Sea, Germany	Kopendorf (Fehmarn Island), Germany	Schleswig, Germany	Kiel, Germany
Sampled Material	jellyfish at the shore of North Sea	surface water from Fjord Schlei	pond surface water	wastewater aeration lagoon of sugar processing plant	water from Schrevenpark pond
**Phenotypic features**					
Pigmentation	lucid-white	dark pink	dark pink	light to dark pink	light to dark pink
Cell Shape	ellipsoidal to pear-shaped	ellipsoidal to pear-shaped	ellipsoidal to pear-shaped	ellipsoidal to pear-shaped	ellipsoidal to pear-shaped
Size (Length x width) (µm)	1.9 ± 0.2 x 1.0 ± 0.2	1.4 ± 0.2 x 1.1 ± 0.2	1.6 ± 0.2 x 1.1 ± 0.2	1.6 ± 0.2 x 1.1 ± 0.2	1.7 ± 0.2 x 1.1 ± 0.2
Cell Division Mode	asymmetric (polar budding)	asymmetric (polar budding)	asymmetric (polar budding)	asymmetric (polar budding)	asymmetric (polar budding)
Temperature Range (optimum) (°C)	10–33 (28)	18–32 (28)	21–28 (28)	18–37 (28)	21–37 (28)
pH Range (optimum)	6.0–8.0 (7.5)	7.0–10.0 (8.0)	6.0–8.0 (7.5)	6.0–10.0 (7.5)	6.0–9.0 (7.5)
Relation to Oxygen	aerobic	aerobic	aerobic	aerobic	aerobic
Stalks	yes	n.o.	n.o.	n.o.	n.o.
Aggregates	yes, rosettes	yes, rosettes	no	no	yes, rosettes

n.o., not observed. Features of *A. helgolandensis* Q31a^T^ were obtained from the respective species description.

### Phenotypic characterization

**Fig. 5:**
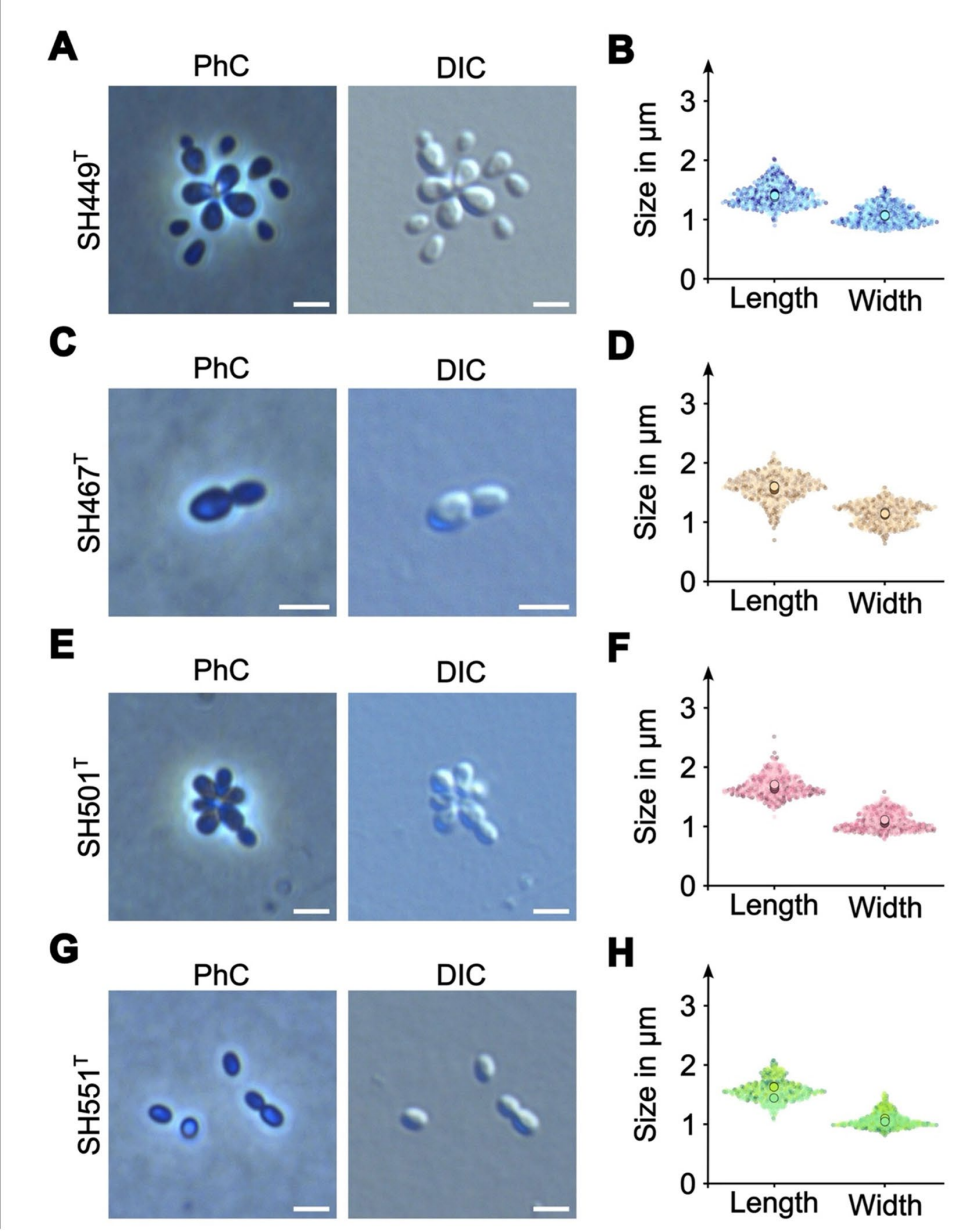
Cell morphology and cell sizes. Phase contrast (PhC) and differential interference contrast (DIC) images of dividing cells of strains SH449^T^ **(A)**, SH467^T^ **(C)**, SH501^T^ **(E)**, and SH551^T^ **(G)**. Smaller daughter cells emerging from larger mother cells are depicted in the early division stage. Cell size analysis of strains SH449^T^ **(B)**, SH467^T^ **(D)**, SH501^T^ **(F)**, and SH551^T^ (H) were determined from three biological replicates. Larger circles indicate mean values of each replicate. The scale bars represent 2 μm.

Microscopically, cells of the analyzed strains were observed to be pear-shaped (Fig. 5A, C, E, G). Out of all four, only strains SH449^T^ and SH501^T^ were able to form rosette-like aggregates (Fig. 5A and E). Cells have a cell length and width in a range of 1.4 to 1.7 μm and 1.1 μm, respectively. Their pear-shaped morphology is a shared feature with their current closest relative *A. helgolandensis* Q31a^T^; however, Q31a^T^ cells were longer, but similar in width^44^. In accordance with strain Q31a^T^ and most members of the order *Pirellulales*^44^, cells of all four strains divide through an asymmetric cell division type (polar budding) in which a small daughter cell emerges on a larger mother cell’s pole (Fig. 5A, C, E, G), grows over time and eventually pinches off from the mother cell.

## Conclusion

In this study, we introduce four novel species belonging to a novel genus in the family *Pirellulaceae*, expanding the known diversity of this understudied bacterial group through high-quality genome sequencing and robust phylogenomic analyses. While the determined genome sequences enabled precise species delineation and genus-level novelty based on five analysed phylogenetic markers, the work relies primarily on these molecular data for taxonomic placement, with physiological and biochemical characterizations limited to standard growth conditions and basic phenotypic traits. Chemotaxonomic profiling including fatty acid, polar lipid, and quinone analyses was not performed, representing a key limitation that precludes deeper insights into cell-wall architecture and membrane adaptations typical of planctomycetes. This approach aligns with the ongoing transition in prokaryotic taxonomy, where maturing genome sequencing technologies increasingly shift species identification toward data accumulation rather than purely phenotypic novelty. Nevertheless, the absence of functional genomic profiling highlights a broader challenge in the field that is predominantly related to the high number of proteins of unknown function encoded in planctomycetotal genomes. Future investigations could leverage these genomes for the analysis of secondary metabolite biosynthesis capabilities, catabolic activities or essential cell biological processes such as cell division to bridge the gap between gene content and phenotypic expression. By explicitly documenting these methodological boundaries, the present work maintains scientific rigor while providing foundational genomic resources that will facilitate subsequent multi-omics and experimental studies. Overall, the description of this novel genus contributes valuable biodiversity data to the broader microbial ecology community, paving the way for integrated taxonomic and functional explorations in the phylum *Planctomycetota*. For the novel taxa, we propose the names *Brakhagea baltica* gen. nov., sp. nov., represented by the type strain SH449^T^, *Brakhagea lacunae* sp. nov., represented by the type strain SH551^T^, *Brakhagea slesvicensis* sp. nov., represented by the type strain SH467^T^, and *Brakhagea aquatica* sp. nov., represented by the type strain SH501^T^.

### Description of *Brakhagea* gen. nov.

*Brakhagea* (Brak.ha’ge.a. N.L. fem. n. *Brakhagea*, named to honor Prof. Axel A. Brakhage for his groundbreaking contributions in microbiology).

Members of the genus are heterotrophic, thrive in aerobic conditions, are mesophilic, and range from neutrophilic to slightly alkaliphilic. Cells are pink-pigmented, pear-shaped (drop-like shape) and divide by polar budding. Genomes have a DNA G + C content in the range of 49–54%. The genus is part of the family *Pirellulaceae*, order *Pirellulales*, class *Planctomycetia*, phylum *Planctomycetota*. The type species of the genus is *Brakhagea baltica*.

### Description of *Brakhageabaltica* sp. nov.

*Brakhagea baltica* (bal’ti.ca. M.L. fem. adj. *baltica*, baltic, referring to its isolation from Fjord Schlei, a part of Baltic Sea).

Cells are ellipsoidal to pear-shaped with an average length and width of 1.4 × 1.1 μm. Cells are aerobic. The type strain is SH449^T^ (= KCTC 102025^T^ = DSM 116586^T^). It was isolated from Fjord Schlei, a 42 km brackish arm of the Baltic Sea. The type strain grows optimally at a pH of 8.0 (range 7.0–10.0) and a temperature of 28 °C (range 18–32 °C). The genome of the type strain is 7.05 Mb in size and has a DNA G + C content of 51.6%. The type strain lacks plasmids.

### Description of *Brakhagea lacunae* sp. nov.

*Brakhagea lacunae* (la.cu’nae. L. gen. n. *lacunae*, of a pond, referring to the isolation from a village pond).

Cells are ellipsoidal to pear-shaped, with an average length and width of 1.6 × 1.1 μm. Cells are aerobic. The type strain is SH551^T^ (= KCTC 102023^T^ = DSM 116785^T^ = CECT 30846^T^). It was isolated from a village pond in Kopendorf (Fehmarn Island), Northern Germany. It grows optimally at a pH of 7.5 (range 6.0–8.0) and a temperature of 28 °C (range 21–28 °C). The genome of the type strain is 7.25 Mb in size with a DNA G + C content of 49.4%. A single plasmid is present.

### Description of *Brakhagea slesvicensis* sp. nov.

*Brakhagea slesvicensis* (sles.vi.cen’sis. M.L. fem. adj. *slesvicensis*, of Slesvicum, the latin name of Schleswig, a city in Northern Germany, from which the type strain was isolated).

Cells are ellipsoidal to pear-shaped, with an average length and width of 1.6 × 1.1 μm. Cells are aerobic. The type strain is SH467^T^ (= KCTC 102026^T^ = DSM 116378^T^). It was isolated from a wastewater aeration lagoon of a sugar processing plant in Schleswig, Northern Germany. It grows optimally at a pH of 7.5 (range 6.0–10.0) and a temperature of 28 °C (range 18–37 °C). The genome of the type strain is 5.87 Mb in size and has a DNA G + C content of 54.2%. The type strain lacks plasmids.

### Description of *Brakhagea aquatica* sp. nov.

*Brakhagea aquatica* (a.qua’ti.ca. L. fem. adj. *aquatica;* pertaining to water, reflecting the isolation from surface water in a lagoon within a pond).

Cells are ellipsoidal to pear-shaped, with an average length and width of 1.7 × 1.1 μm. Cells are aerobic. The type strain is SH501^T^ (= KCTC 102088^T^ = CECT 30906^T^). It was isolated from Schrevenpark pond (Kiel) in Northern Germany. The type strain grows optimally at a pH of 7.5 (range 6.0–9.0) and a temperature of 28 °C (range 21–37 °C). The genome of the type strain is 6.70 Mb in size, has a DNA G + C content of 53.1% and includes a plasmid.

## Supplementary Information

Below is the link to the electronic supplementary material.

Supplementary Material 1

Supplementary Material 2

## Data Availability

The 16 S rRNA gene sequences were deposited in the GenBank database under the following accession numbers: PV955651 (SH449^T^), PV955658 (SH551^T^), PV955674 (SH467^T^) and PV955675 (SH501^T^). Genome sequence information is available from NCBI under the accession numbers: SH449^T^: CP197409; SH551^T^: CP197424 (chromosome) and CP197425 (plasmid pSH551_1); SH467^T^: CP197412; SH501^T^: CP197422 (chromosome) and CP197423 (plasmid pSH501_1).
